# Supplementary material for: Learning to detect AI texts and learning the limits
Source: PLoS One. 2025 Oct 15;20(10):e0333007. doi: 10.1371/journal.pone.0333007 (PMC12527182; doi:10.1371/journal.pone.0333007)
Supplement: S2 File — All data and scripts used in the study, some additional descriptive statistics, scalable formate figures. (ZIP) [file pone.0333007.s002.tgz › data and scripts/OSF/scripts/analysis/PCA/eigenvectors_chart.pdf]

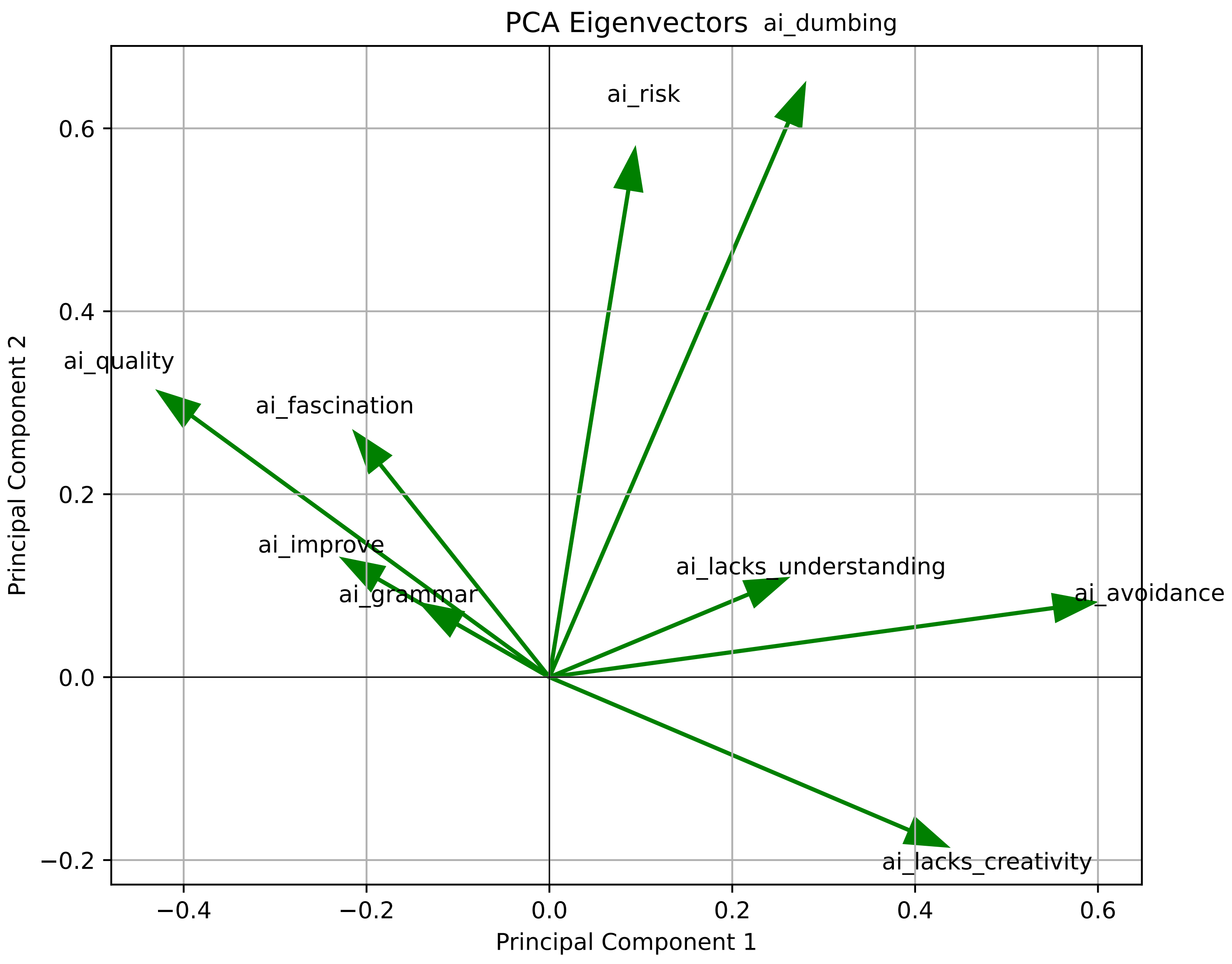

PCA Eigenvectors
ai_dumbing
ai_risk
ai_quality
ai_fascination
ai_improve
ai_grammar
ai_lacks_understanding
ai_avoidance
ai_lacks_creativity
0.6
0.4
0.2
0.0
−0.2
−0.4
−0.2
0.0
0.2
0.4
0.6
Principal Component 1
Principal Component 2
